# Combined NK-CIK and PD-1 inhibitor (nivolumab), an effective immunotherapy for treating intrahepatic lymphoepithelioma-like cholangiocarcinoma unassociated with EBV infection: Two case reports and a literature review

**DOI:** 10.3389/fonc.2023.1090580

**Published:** 2023-01-30

**Authors:** Alen Sam Saji, Biao Yang, Wan Ting Hou, Xia Liu, Qiu Ping Ren, Yuan Feng Wei, Yu Zu Zhang, Xi Yang

**Affiliations:** ^1^ Abdominal Oncology Ward, Cancer Center, West China Hospital, West China Medical School, Sichuan University, Chengdu, China; ^2^ Department of Gastroenterology, West China Hospital, West China Medical School, Sichuan University, Chengdu, China; ^3^ Department of Liver Surgery, West China Hospital, West China Medical School, Sichuan University, Chengdu, China

**Keywords:** liver, lymphoepithelioma-like carcinoma, cholangiocarcinoma, EBV, nivolumab

## Abstract

Intrahepatic lymphoepithelioma-like cholangiocarcinoma (LELCC) is a very rare malignant tumor arising from the biliary epithelium. To date, there has been a lack of evidence on the radiographical features, clinicopathological features, and treatment modalities of LELCC, with less than 28 cases of LELCC without Epstein–Barr virus (EBV) infection having been reported worldwide. The treatment of LELCC remains unexplored. Here, we present two cases of patients with LELCC without EBV infection who were treated by liver resection, chemotherapy, and immunotherapy and who achieved long survival time. The patients received surgery to remove the tumors and then adjuvant chemotherapy using the GS regimen and combined immunotherapy involving natural killer–cytokine-induced killer (NK-CIK) and nivolumab were performed. Both patients had a good prognosis with a survival time of more than 100 months and 85 months.

## Introduction

Intrahepatic lymphoepithelioma-like carcinoma (LELCC) is a very rare tumor that is composed of undifferentiated carcinoma with a high lymphocytic infiltrate that is morphologically similar to undifferentiated nasopharyngeal carcinoma and is often accompanied by Epstein–Barr virus (EBV) infection (1). The clinicopathological features are indefinite and variable; thus, appropriate imaging techniques should be done alongside for a definitive diagnosis (2). However, LELCC cases without EBV infection are less common (1, 3–5). Hence, clinical researchers around the world are constantly exploring various therapeutic methods to treat LELCC. Currently, the common treatment options for LELCC include liver resection, chemotherapy, immunotherapy, radiofrequency ablation, and microwave ablation. Often, most of these treatments are accompanied by unfavorable side effects; thus, it is important for regular post-treatment follow-up (6). Here, we provide a potential effective treatment for two cases of LELCC without EBV infection using combined immunotherapy using nivolumab and NK-CIK; the treatment plan was determined after carefully examining the literature for protocols against similar tumors. Furthermore, we reviewed the radiography, clinicopathological features, and treatment options of LELCC.

## Case presentation

### Case 1

**Figure 1:**
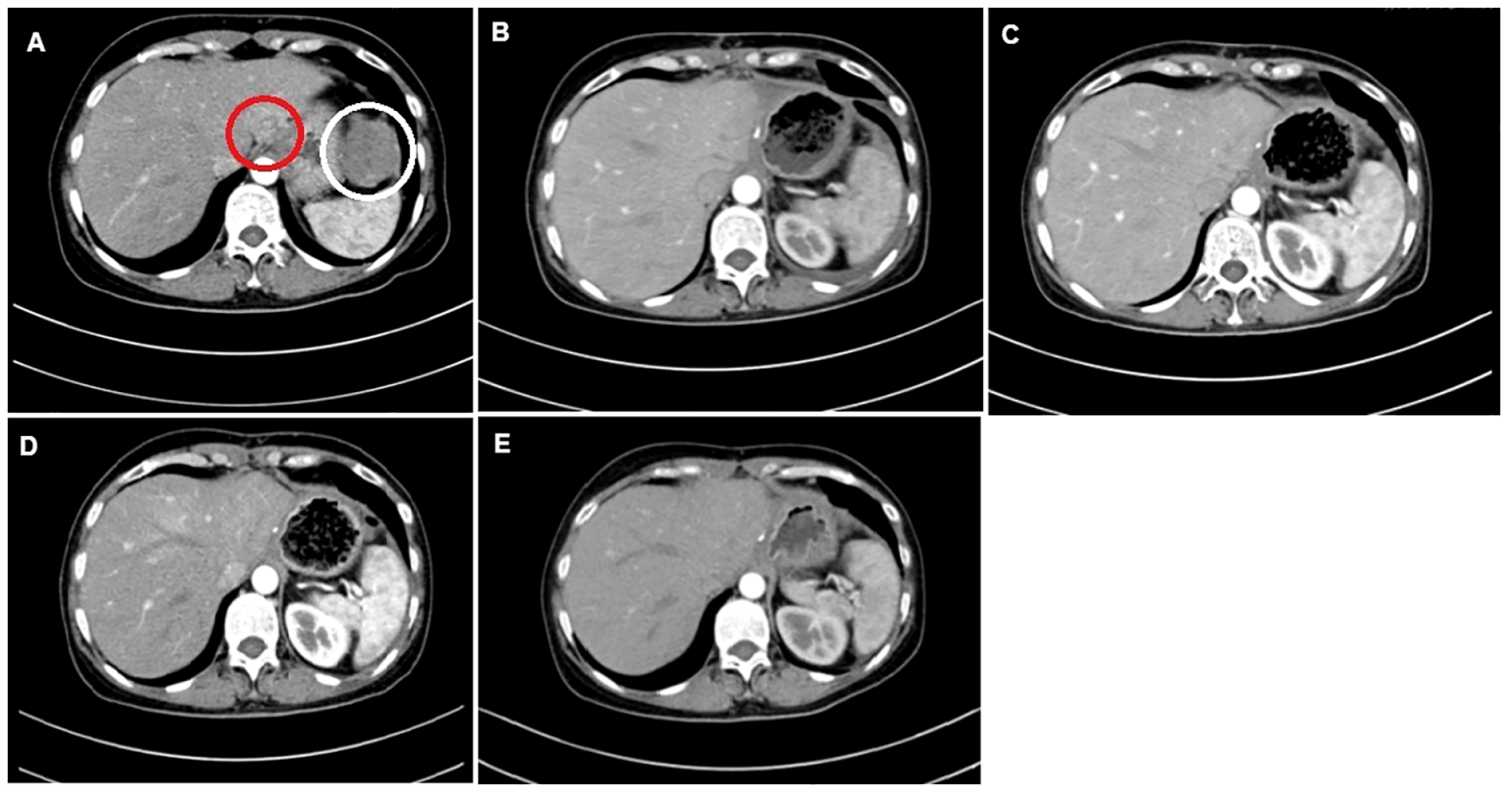
CT images of the 53-year-old female patient. **(A)** Tumor lesions of size 4.9 × 3.6 cm (red circle) and 7 × 6 cm (white circle). **(B)** Post-operative CT image before the first cycle of the GS regimen. **(C)** CT image post-chemotherapy after the second cycle of the GS regimen. **(D)** CT evaluation post-chemotherapy after the fifth cycle of the GS regimen. **(E)** CT evaluation post-chemotherapy after the sixth cycle.

Eight years ago, a 53-year-old female patient was transferred to our hospital as multiple lesions that were misdiagnosed as atypical hemangiomas were found in the liver. The patient presented with an increase in alpha fetoprotein (AFP) and severe right upper quadrant pain. She underwent a CT scan (**Figure 1**), which revealed multiple lesions in the left lobe of the liver, the left ovarian region, and the intraperitoneal region above the spleen. Hence, laparoscopic exploration and bilateral abdominal hysterectomy and adnexectomy were performed. During the operation, a new lesion (4 cm) was found at the gastric cardia. The pathological examination showed mature teratoma of the left ovary combined with metastatic adenocarcinoma. Two LELCC tumors of size 4.9 cm × 3.6 cm and 7 cm × 6 cm were present at the edge of the left lateral lobe of the liver and anterior to the spleen, respectively. The tumors were hard with white nodular changes and quick-freezing results showed adenocarcinoma, which was non-hepatocellular. Then, resection of metastatic gastric tumor, resection of liver tumor, and diaphragmatic tumor resection were performed. Serum level testing revealed the following results: hepatitis B surface antigen (HBsAg) (+), hepatitis C virus (HCV) (−), and EBV (−). Immunohistochemical staining results showed that the tumor cells were cytokeratin (CK7) (–), CK18 (–), CK19 (+), CK20 (+), HepPar-1(–), CA199 (–), CA125 (–), p53 gene (–), human epidermis growth factor receptor (HEGF) (+), and HER-2 (+); the positive rate of cell proliferation-related antigen Ki-67 was about 20% to 30% and EBV-encoded RNA 1 or 2 *in situ* hybridization (−). Other tumor markers were as follows: AFP, 86.40 ng/ml; carcinoembryonic antigen (CEA), 1.41 ng/ml; and carbohydrate antigen (CA), 199 0.94 U/ml. Three months later, enhanced CT showed new lesions in the omentum and pelvic cavity. There was also lymph node metastasis in the intraperitoneal, retroperitoneal, right external iliac, and right inguinal regions. She received chemotherapy with gemcitabine (GS) regimen (gemcitabine dose of 400 mg/day IV on day 1 and day 8: S-1 at a dose of 50 mg/time, orally, two times/day, from day 1 to day 14 for six cycles of chemotherapy). Then, the patient received four cycles of NK-CIK cells (autologous blood) and nivolumab at 150 mg (3 mg/kg) per dose.

After the second, fourth, and sixth cycles of chemotherapy, the chest and abdomen were re-examined with enhanced CT scan, and the efficacy evaluation was stable disease. One year later, no new tumors were found. There were no signs of tumor recurrence and metastasis. The patient was followed up regularly and is currently alive after 100 months.

### Case 2

**Figure 2:**
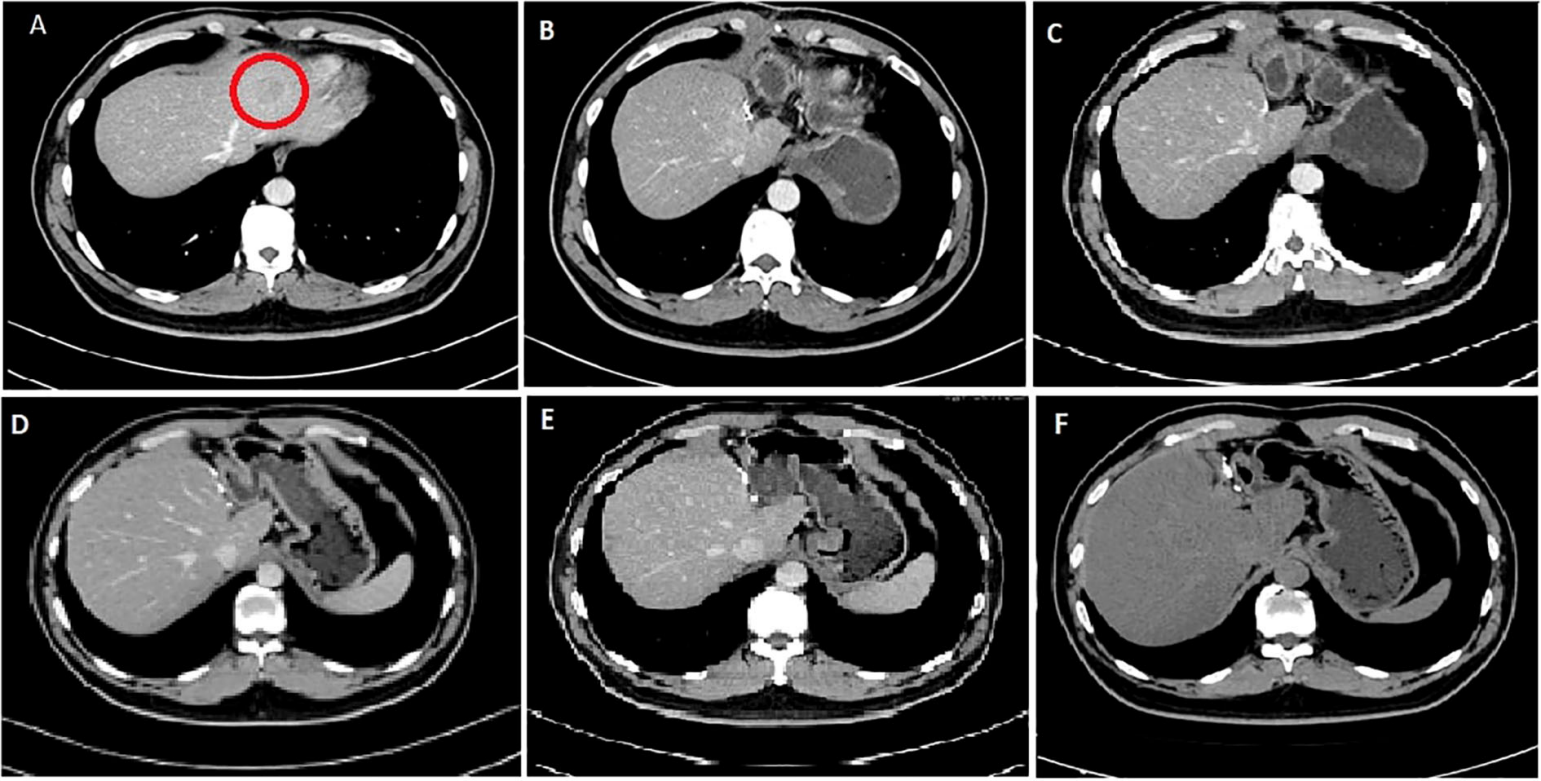
CT images of the 41-year-old male patient. **(A)** First diagnostic CT image showing tumor lesion (red circle) of size 3.0 × 2.8 cm taken in 2015. **(B)** CT image post-operation and after the first cycle of the GS regimen; no signs of tumor recurrence or metastasis. **(C)** CT image after the second cycle of the GS regimen. **(D)** CT image after the fourth cycle of the GS regimen. **(E)** CT image after the sixth cycle of the GS regimen. **(F)** CT image taken on follow-up in 2022.

Seven years ago, a 41-year-old male patient was referred to West China Hospital due to a left hepatic mass found during physical examination. CT scan revealed a low-density shadow of approximately 3.0 cm × 2.8 cm in the left lateral lobe (**Figure 2**), with unclear boundaries, irregular shape, and uneven edges seen on enhancement. There were no signs of lymph node metastasis. Routine blood test on admission showed the following: red blood cells, 4.92 × 10^12^/L; hemoglobin, 155 g/L; and platelet count, 179×10^9^/L. Biochemical tests revealed the following: total bilirubin, 15.9 μmol/L; ALT, 56 U/L; AST, 28 U/L; and albumin, 48.9 g/L. Routine coagulation examination showed a prothrombin time of 11.4 s. Tumor marker examination revealed the following: AFP, 3.10 ng/ml; CEA, 0.64 ng/ml; CA199, 51.95 U/ml; and CA125, 8.38 U/ml. Serum testing showed the following: HBsAg (+), hepatitis B e-antigen (HBeAg) (+), and hepatitis B core antigen (HBcAg) (+).

**Figure 3:**
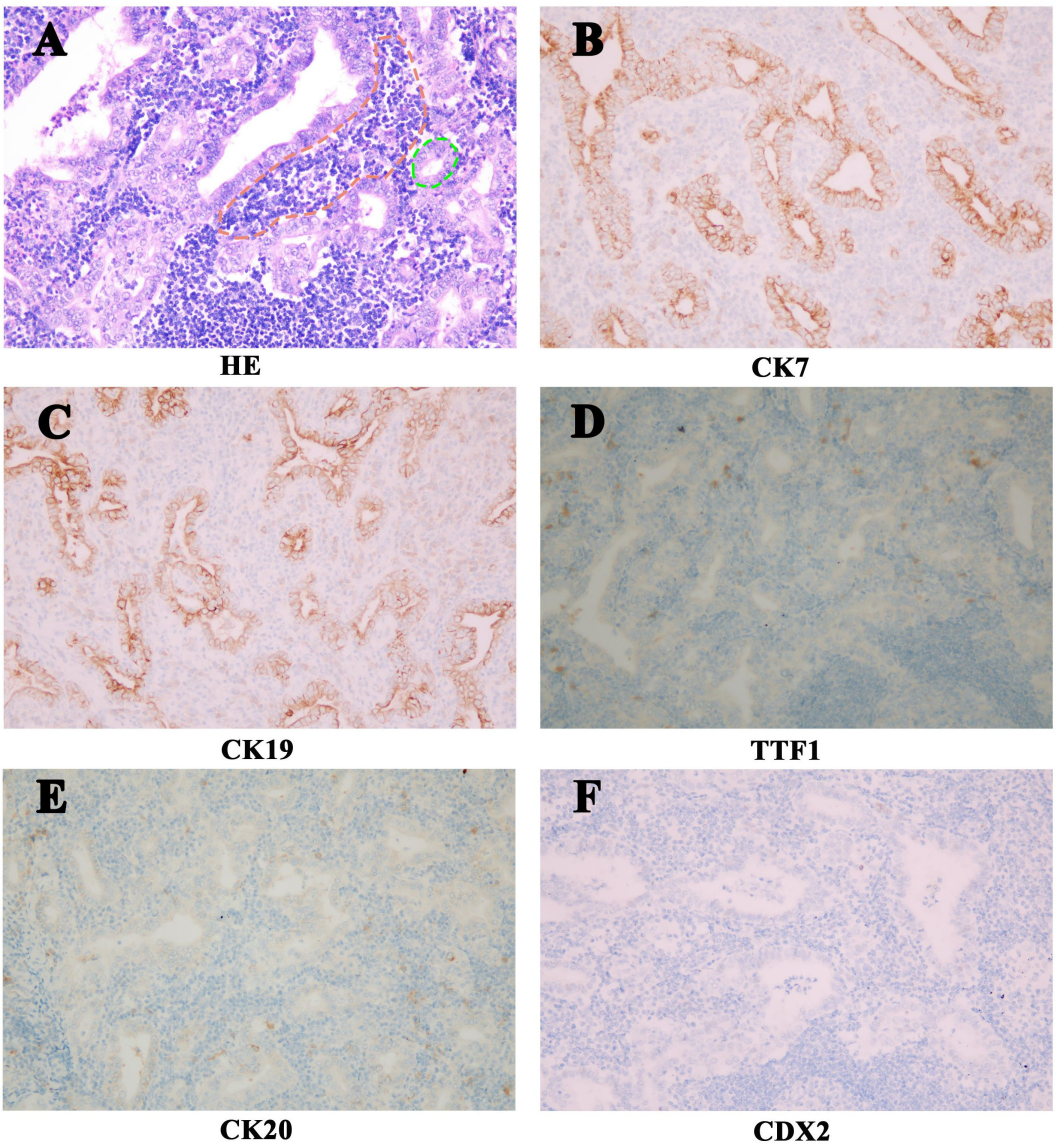
The Histologic features of the LELCC. **(A)** Hematoxylin - eosin staining (HE, 200×) shows poorly differentiated bile duct cells (Green) with lymphocytic infiltrates (Red). Immunohistochemically (200×), the tumor cells showed **(B)** CK7 and **(C)** CK19 expression, while no expression of **(D)** TTF1, **(E)** CK20 or **(F)** CDX2 (200×).

Then, an operation was performed, during which a tumor with a diameter of 3 cm was located on the left lateral lobe of the liver and was resected. The left liver had moderately differentiated adenocarcinoma, with more lymphocytes and some plasma cells infiltrated in the interstitium. Immunohistochemical staining results showed the following: tumor cells CK7 (+), CK19 (+), caudal related homeobox (CDX2) (–), thyroid transcription factor (TTF-1) (–), CK20 (–) (**Figure 3**), and *in situ* hybridization of EBV-encoded RNA 1 or 2 (EBV-encoded RNA 1/2, EBER1/2) (−); it was considered to be EBV-negative LELCC. The real-time fluorescence detection of EBV DNA was negative, EBV capsid antigen IgA antibody was negative, and EBV early antigen IgG antibody was negative. Six months later, as he had a recurrence, he received an adjuvant chemotherapy with the GS regimen for six cycles followed by four doses of NK-CIK cells and nivolumab treatment (240 mg per time).

After the second and fourth cycles, the chest and abdomen CT showed no signs of tumor recurrence and metastasis. After the sixth cycle, re-examination of EBV DNA real-time fluorescence detection showed negative results; positron emission tomography showed no signs of tumor recurrence in the whole body. With regular follow-up, the patient is currently alive after 85 months post-treatment.

## Discussion

LELCC is an extremely rare subtype of cholangiocarcinoma originating from the epithelium and is a poorly differentiated carcinoma; its typical histological findings consist of pleomorphic tumor cells with many mature small lymphocyte infiltrations (7). The primary site of LELCC is in the nasopharynx, but there are other organs and tissues found to be capable of exhibiting malignant growth, but LELCC in the liver is a rare occurrence (1).

Since the first reported LELCC in 1996 by Hsu et al., there were 113 cases that have been reported (8) (**Supplementary Table 1**). The majority of the reported cases were among Asians (*n* = 108, 95.5%). The median age was 55 years, and there seems to be a higher prevalence in female patients than in male patients (F:M = 70:43); 75.2% (85/113) of the patients have an EBV infection, and only 24.7% (28/113) have no EBV infection. The HBV infection rate was 49.3%, the rate of HCV infection was 9.73%, and the presence of cirrhosis was 9.73%.

According to the literature, the size of the tumor varies from 0.8 cm to 12 cm. Patients with LELLC are diagnosed with a large tumor size due to the varying symptoms or unspecific imaging and laboratory tests (9). As of now, a total of 70 patients have presented immunohistochemical features (**Supplementary Table 2**). Among these patients, 62.8% had positive expression of CK7, 70% had positive expression of CK19, and 57% had no expression of HepBar-1, which supports the findings of another study (10) and may be an important feature in diagnosis. High expression of CK7 and CK19 suggests that the origin of the tumor could be from the biliary epithelium (11). Other less common markers that were reported in the literature include CK8, CK18, CK20, IDH1, LMP1, CEA, AFP, CDX2, CA-199, HER-2, TTF-1, and MNF116 (**Supplementary Table 2**).

A total of 36 cases have reported radiological characteristics (**Supplementary Table 3**). Considerable enhancement and washout were reported in the arterial phase, and it supported the hypervascular characteristic of LELCC (12). It was noted that the characteristics of LELCC were highly variable from each other, and the diagnosis would not be possible with the imaging features alone (9). It was also noted that there was a higher incidence of LELCC in the right lobe than in the left (R:L = 58:45).

LELCC has a good prognosis according to the available literature; even cases with mixed pathological type or locally advanced cases of LELCC with local recurrence and distant metastasis may still have long-term survival (5). The reported treatment protocol included liver resection in 77.8% of patients, chemotherapy in 9.7%, and radiofrequency ablation in 3.5%. Recently, Li et al. (9) reported a case of LELCC that was treated with microwave ablation that achieved patient overall survival for 9 months. This study revealed that 63.71% of the reported cases were alive with no signs of disease, while 15.9% were alive with neoplastic disease and 17.6% died during interventions or post-surgical complications. The reported follow-up period for patients who were cured varied from 2 months to 165 months (1, 13). Studies have suggested the relationship of PD-1 receptor as a potential therapeutic target site for LELCC as it is upregulated in LELCC patients (10), and several studies also highlight the efficiency of NK cells in targeting and eliminating cancerous cells as they are innate cytotoxic lymphocytes and cytokines that are vital regulators of NK cells and are very appealing to stimulate an anticancer response (14). For the patients in the current series, NK-CIK was administered through autologous blood transfusion, and both patients did not experience any adverse side effects. In summary, we analyzed all the cases of LELCC published in the literature and we were able to achieve a good prognosis with the combined immunotherapy treatment using PD-1 inhibitor and NK-CIK.

## Data availability statement

The original contributions presented in the study are included in the article/**Supplementary Material**. Further inquiries can be directed to the corresponding authors.

## Author contributions

AS and BY conceived and designed the study. AS and XL wrote the initial manuscript. WH and YZ were responsible for data collection and acquisition. QR and YW were responsible for the histopathological analysis. AS, BY and XY critically revised the manuscript. All authors contributed to the article and approved the submitted version.
