# Supplementary material for: Combined NK-CIK and PD-1 inhibitor (nivolumab), an effective immunotherapy for treating intrahepatic lymphoepithelioma-like cholangiocarcinoma unassociated with EBV infection: Two case reports and a literature review
Source: Front Oncol. 2023 Jan 30;13:1090580. doi: 10.3389/fonc.2023.1090580 (PMC9971717; doi:10.3389/fonc.2023.1090580)
Supplement: Supplementary file 1 [file Table_1.docx]

Supplementary Table 1: Summary of clinical features of ICC patients with LELCC ^[1-35]^

| No. | Study/  Author | Year | Ethnicity | Sex / Age | Tumor location | Size  (cm) | Treatment | HBV | HCV | EBV | Metastasis  (Absent/  Present) | Cirrhosis | Outcome (Follow-up, months) |
| --- | --- | --- | --- | --- | --- | --- | --- | --- | --- | --- | --- | --- | --- |
| 1 | **Current study (n=2)** | 2022 | Asian  (n=2) | F/53 | LL | 7* | LR + CT (GS-1) | + | + | - | Present | Absent | AWND (100) |
| 2 |  |  |  | M/41 | LL | 3 | LR + CT (GS-1) | + | + | - | Absent | Absent | AWND (85) |
| 3 | **Henderson-Jackson et al. ^[10]^ (n=1)** | 2010 | Asian  (n=1) | F/63 | LL | 4 | LR | - | - | + | Absent | Absent | AWND (6) |
| 4 | **Hsu et al. ^[9]^**  **(n=1)** | 1996 | Asian  (n=1) | F/47 | RL | 12 | LR | - | - | + | Present | Absent | DOD (NA) |
| 5 | **Binny et al. ^[7]^**  **(n=3)** | 2022 | Asian  (n=3) | F/30 | NA | 2.3 | LR | + | - | + | Absent | Absent | AWND (22) |
| 6 |  |  |  | M/71 | NA | 1.1 | LR | + | - | + | Absent | Absent | AWND (30) |
| 7 |  |  |  | F/72 | NA | 2.6 | LR | + | - | + | Absent | Absent | AWND (62) |
| 8 | **Jeng et al. ^[8]^ (n=**4**)** | 2001 | Asian  (n=4) | M/42 | RL | 3 | LR | - | - | + | Absent | Absent | AWND (84) |
| 9 |  |  |  | F/67 | LL | 3 | LR | - | - | + | Absent | Absent | AWND (7) |
| 10 |  |  |  | M/50 | RL | 4 | LR | + | - | + | Absent | Absent | AWND (16) |
| 11 |  |  |  | F/50 | LL | 4 | LR | + | - | + | Absent | Absent | AWND (2) |
| 12 | **Wang et al. ^[3]^**  **(n=13)** | 2016 | Asian  (n=13) | M/60 | RL | 6 | LR | + | - | + | Absent | Absent | DOD (27) |
| 13 |  |  |  | M/53 | RL | 2.4 | LR | - | - | + | Absent | Absent | DOD (22) |
| 14 |  |  |  | M/39 | RL | 2 | LR | - | - | - | Absent | Absent | AWND (54) |
| 15 |  |  |  | F/43 | RL | 1.2 | LR | - | - | + | Absent | Absent | AWND (51) |
| 16 |  |  |  | M/62 | RL | 3.2 | LR | + | - | - | Absent | Absent | AWND (37) |
| 17 |  |  |  | F/65 | RL | 5 | LR | + | - | - | Absent | Absent | AWND (37) |
| 18 |  |  |  | F/47 | LL | 1.9 | LR | - | - | + | Absent | Absent | AWND (35) |
| 19 |  |  |  | M/52 | RL | 1.9 | LR | + | - | - | Absent | Absent | AWND (19) |
| 20 |  |  |  | M/60 | LL | 2.6 | LR | - | - | + | Absent | Absent | AWND (12) |
| 21 |  |  |  | M/61 | RL | 2.4 | LR | + | - | - | Absent | Absent | AWD (12) |
| 22 |  |  |  | M/35 | RL | 3.1 | LR | + | - | + | Absent | Absent | AWND (10) |
| 23 |  |  |  | M/48 | RL | 3.5 | LR | - | - | + | Absent | Absent | AWND (9) |
| 24 |  |  |  | F/71 | RL | 2.2 | LR | - | - | + | Absent | Absent | AWND (8) |
| 25 | **Sun et al. ^[4]^**  **(n=9)** | 2016 | Asian  (n=9) | F/35 | RL | 2 | NA | + | - | + | Absent | Absent | AWND (69) |
| 26 |  |  |  | M/58 | LL | 2.9 | NA | - | - | + | Present | Absent | DOD (23) |
| 27 |  |  |  | M/54 | RL | 3.7 | NA | + | - | + | Present | Absent | DOD (48) |
| 28 |  |  |  | F/52 | LL | 4.1 | NA | - | - | + | Present | Absent | DOD (53) |
| 29 |  |  |  | M/54 | RL | 1.8 | NA | + | - | + | Absent | Present | AWD (45) |
| 30 |  |  |  | F/30 | RL | 3 | NA | + | - | + | Absent | Absent | AWND (35) |
| 31 |  |  |  | F/67 | RL | 3 | NA | - | - | + | Absent | Absent | NA |
| 32 |  |  |  | F/51 | RL | 3.2 | NA | + | - | + | Absent | Absent | NA |
| 33 |  |  |  | F/63 | RL | 3.1 | NA | - | - | + | Absent | Absent | AWD (24) |
| 34 | **Gearty et al. ^[11]^ (n=1)** | 2019 | Asian  (n=1) | F/28 | LL | 6.8 | CT | + | - | + | Present | Absent | DOD (9) |
| 35 | **Adachi et al. ^[14]^**  **(n=1)** | 2008 | Asian  (n=1) | M/64 | LL | 5.2 | LR | - | - | - | Absent | Absent | AWND (3) |
| 36 | **Chen et al. ^[35]^**  **(n=2)** | 2001 | Asian  (n=2) | F/67 | RL | 5 | LR | - | + | + | Present | Absent | DOD (<1) |
| 37 |  |  |  | M/41 | LL | 3 | LR | + | - | - | Absent | Present | AWND (8) |
| 38 | **Hur et al. ^[32]^**  **(n=1)** | 2011 | Asian  (n=1) | F/57 | RL | 2 | LR | - | - | - | Absent | Absent | AWND (60) |
| 39 | **Ishida et al. ^[31]^**  **(n=1)** | 2011 | Asian  (n=1) | M/68 | CBD | 3 | PAD | - | + | - | Absent | NA | AWND (5) |
| 40 | **Kim et al. ^[30]^**  **(n=1)** | 1999 | Asian  (n=1) | M/64 | RL | 2 | LR | - | + | - | Absent | Present | AWND (NA) |
| 41 | **Labgaa et al. ^[6]^ (n=1)** | 2017 | Asian  (n=1) | M/58 | RL+ LL | 2.2 | LR | + | - | + | Absent | Absent | AWND (61) |
| 42 | **Lee ^[29]^**  **(n=1)** | 2011 | Asian  (n=1) | M/79 | RL | 3.5 | LR + CT | + | - | - | Present | Present | AWND (54) |
| 43 | **Liao et al. ^[28]^**  **(n=1)** | 2015 | Asian  (n=1) | F/35 | LL | 1.7 | LR | + | - | + | Present | Present | AWND (NA) |
| 44 | **Ortiz et al. ^[23]^**  **(n=1)** | 2000 | Caucasian  (n=1) | F/19 | LL | 5.5 | LR | - | - | + | Absent | Absent | DOD (44) |
| 45 | **Szekely ^[22]^**  **(n = 1)** | 2001 | Asian  (n=1) | M/61 | NA | 6 | LR | - | - | - | Absent | Absent | AWND (11) |
| 46 | **Vortmeyer et al. ^[18]^ （n=1）** | 1997 | Caucasian  (n=1) | F/71 | Porta-Hepatis | 5 | NA | - | - | + | Absent | Absent | AWD (36) |
| 47 | **Chan et al. ^[12]^**  **(n = 7)** | 2014 | Asian  (n=7) | F/53 | RL | 1.6 | LR | + | - | + | Absent | Present | AWND (165) |
| 48 |  |  |  | F/40 | RL | 7.5 | LR | + | - | + | Absent | Absent | AWD (56) |
| 49 |  |  |  | F/57 | LL | 7.1 | LR | - | - | + | Absent | Absent | AWND (128) |
| 50 |  |  |  | F/56 | LL | 6 | LR | - | - | + | Absent | Absent | DOD (69) |
| 51 |  |  |  | F/59 | LL | 6 | LR | + | - | + | Absent | Absent | AWND (72) |
| 52 |  |  |  | F/45 | LL | 3 | LR | - | - | + | Absent | Absent | AWD (71) |
| 53 |  |  |  | F/57 | RL | 3 | LR | - | - | + | Absent | Absent | AWND (58) |
| 54 | **Aosasa** **et al. ^[13]^ (n=1)** | 2015 | Asian  (n=1) | F/65 | LL | 6.4 | LR | - | + | - | Present | Absent | AWND (20) |
| 55 | **Min et al. ^[25]^**  **(n=1)** | 2007 | Asian  (n=1) | M/46 | NA | 2.7 | LR | + | - | + | Absent | Absent | AWD (84) |
| 56 | **Ding et al. ^[34]^**  **(n=1)** | 2019 | Asian  (n=1) | F/75 | LL | 1.5 | LR | - | - | + | Present | Absent | AWND (3) |
| 57 | **Lin et al. ^[27]^**  **(n=2)** | 2020 | AA  (n=2) | F/61 | RL | 2.3 | NA | - | - | + | Present | Present | DOD (NA) |
| 58 |  |  |  | M/23 |  | 6.6 | NA | - | - | + |  | Absent | NA |
| 59 | **Huang et al. ^[33]^**  **(n=9)** | 2021 | Asian  (n=9) | F/46 | LL | 4.5 | LR | - | - | + | NA | Absent | AWND (36) |
| 60 |  |  |  | F/60 | RL | 2.3 | LR | + | - | + | NA | Absent | AWND (44) |
| 61 |  |  |  | F/43 | RL | 5.5 | LR+CT | + | - | + | Present | Absent | DOD (22) |
| 62 |  |  |  | F/43 | LL | 2.5 | LR | + | - | + | NA | Absent | AWND (38) |
| 63 |  |  |  | F/47 | RL | 3 | LR | - | - | + | Absent | Absent | AWND (38) |
| 64 |  |  |  | F/62 | LL | 2.5 | LR+CT+ IT+RT | - | - | + | NA | Absent | AWND (41) |
| 65 |  |  |  | M/54 | LL | 3.5 | LR | - | - | + | Absent | Absent | AWND (37) |
| 66 |  |  |  | F/54 | LL | 1 | LR | + | - | + | NA | Absent | AWND (49) |
| 67 |  |  |  | F/56 | RL | 3 | LR + CT | + | - | + | Absent | Absent | AWND (44) |
| 68 | **Yang et al. ^[2]^**  **(n=11)** | 2021 | Asian  (n=11) | F/47 | RL | 2.8 | LR | - | - | + | Absent | NA | AWND (32.5) |
| 69 |  |  |  | F/61 | RL | 2.6 | LR | + | - | + | Absent | NA | AWND (13.1) |
| 70 |  |  |  | F/60 | RL | 2.5 | LR | + | - | + | Absent | NA | AWND (38.9) |
| 71 |  |  |  | F/30 | LL | 3 | LR | + | - | + | Present | NA | AWD (13.4) |
| 72 |  |  |  | F/53 | LL | 1.1 | LR | + | - | + | Absent | NA | AWND (45.2) |
| 73 |  |  |  | F/33 | RL | 2.1 | RFA | + | - | + | Absent | NA | AWND (19) |
| 74 |  |  |  | F/43 | LL | 2.8 | LR | + | - | + | Absent | NA | AWND (33) |
| 75 |  |  |  | M/54 | LL | 4.5 | LR | + | - | + | Absent | NA | AWND (44.7) |
| 76 |  |  |  | F/55 | LL | 6.7 | CT with GS-1 | - | - | + | Present | NA | AWD (26.6) |
| 77 |  |  |  | F/63 | LL | 7.3 | PD-1 inhibitor | - | - | + | Present | NA | AWD (14.7) |
| 78 |  |  |  | F/55 | RL | 8.7 | CT with GS-1 | + | - | + | Present | NA | AWD (41.7) |
| 79 | **Tai et al. ^[21]^**  **(n=1)** | 2015 | Asian  (n=1) | M/56 | LL | 2.4 | LR | - | - | + | Absent | NA | AWND (3) |
| 80 | **Tsai et al. ^[19]^ (n=25)** | 2021 | Asian (n=25) | M/54 | RL | 0.8 | LR | + | - | - | NA | Absent | AWND (NA) |
| 81 |  |  |  | F/53 | RL | 3.5 | LR | + | - | + | NA | Absent | AWND (NA) |
| 82 |  |  |  | F/72 | RL | 3.0 | LR | - | + | - | NA | Present | AWD (NA) |
| 83 |  |  |  | M/70 | RL | 1.0 | LR | - | + | - | NA | Absent | AWND (NA) |
| 84 |  |  |  | M/53 | LL | 2.0 | LR | NA | NA | + | NA | Absent | AWND (NA) |
| 85 |  |  |  | M/66 | RL | 5.5 | LR | + | - | - | NA | Present | DOD (NA) |
| 86 |  |  |  | M/72 | RL | 5.5 | LR | - | - | + | NA | Absent | AWD (NA) |
| 87 |  |  |  | F/57 | LL | 5.2 | LR | + | - | - | NA | Present | DOD (NA) |
| 88 |  |  |  | M/68 | RL | 6.0 | LR | + | - | - | NA | Absent | DOD (NA) |
| 89 |  |  |  | F/51 | LL | 7.5 | LR | - | - | + | NA | Absent | DOD (NA) |
| 90 |  |  |  | F/63 | LL | 11.8 | LR | - | - | + | NA | Absent | DOD (NA) |
| 91 |  |  |  | F/54 | LL | 5.0 | LR | + | - | + | NA | Absent | AWND (NA) |
| 92 |  |  |  | F/74 | LL | 4.0 | LR | - | - | + | NA | Absent | AWD (NA) |
| 93 |  |  |  | F/48 | RL | 2.3 | RFA | + | - | + | NA | Absent | AWND (NA) |
| 94 |  |  |  | F/62 | RL | 4.5 | LR | NA | - | - | NA | Present | AWD (NA) |
| 95 |  |  |  | F/64 | RL | 10.0 | LR | - | - | + | NA | Absent | DOD (NA) |
| 96 |  |  |  | F/77 | RL | 4.6 | CT | - | + | + | NA | Absent | AWD (NA) |
| 97 |  |  |  | M/52 | LL | 2.0 | LR | + | - | - | NA | Absent | AWND (NA) |
| 98 |  |  |  | F/68 | RL | 2.2 | LR | - | - | + | NA | Absent | AWND (NA) |
| 99 |  |  |  | M/68 | RL | 2.5 | LR | - | - | - | NA | Absent | DOD (NA) |
| 100 |  |  |  | F/66 | LL | 3.7 | LR | - | + | - | NA | Absent | AWND (NA) |
| 101 |  |  |  | F/64 | LL | 6.0 | LR | - | - | + | NA | Absent | AWD (NA) |
| 102 |  |  |  | F/65 | RL | 11.5 | CT+RT | - | - | + | NA | Absent | AWD (NA) |
| 103 |  |  |  | M/77 | RL | 1.7 | LR | + | - | - | NA | Absent | AWND (NA) |
| 104 |  |  |  | M/43 | RL | 2.3 | RFA | + | - | + | NA | Absent | AWND (NA) |
| 105 | **Zhang et al. ^[15]^ (n=1)** | 2018 | Asian  (n=1) | F/41 | RL | 2.8 | LR | NA | NA | + | Present | NA | AWND (9) |
| 106 | **Xiao et al. ^[16]^**  **(n=1)** | 2015 | Asian  (n=1) | F/62 | RL | 8 | LR | + | - | + | Absent | Absent | AWND (6) |
| 107 | **Nogami et al. ^[24]^ (n=1)** | 2021 | Asian  (n=1) | F/27 | RL | 8 | RFA | + | - | + | Absent | Absent | AWND (12) |
| 108 | **Li et al. ^[5]^**  **(n=1)** | 2022 | Asian  (n=1) | M/55 | RL | 5.1 | MWA | - | + | + | NA | Absent | AWND (9) |
| 109 | **Ling et al. ^[26]^**  **(n=2)** | 2019 | Asian  (n=2) | M/64 | LL | 2 | LR | + | - | + | Absent | Absent | AWND (11) |
| 110 |  |  |  | M/40 | LL | 3 | LR | + | - | + | Absent | Absent | AWND (32) |
| 111 | **Wei et al. ^[17]^**  **(n=1)** | 2015 | Asian  (n=1) | F/42 | LL | 4.7 | LR | + | - | - | Present | Absent | AWND (NA) |
| 112 | **Tan et al. ^[20]^**  **(n=1)** | 2017 | Caucasian  (n=1) | M/22 | NA | NA | CT | - | - | + | Present | NA | DOD (NA) |
| 113 | **Zhang K et al. ^[1]^ (n=1)** | 2020 | Asian  (n=1) | M/62 | LL | 2.8 | LR | + | - | - | Absent | Absent | AWND (15) |

AWND - Alive with no evidence of disease; AWD - Alive with Disease; DOD – Dead of Disease, LR – Liver resection; GS-1- Gemcitabine plus S-1; RFA - Radiofrequency Ablation; EBV – Epstein Barr Virus; HBV- Hepatitis B virus; HCV- Hepatitis C virus; PAD - Pancreaticoduodenectomy; CBD- Common Bile duct; IR- Immunotherapy; RT- Radiotherapy; RFA - radio frequency ablation; MWA- Microwave ablation; LL- Left lobe; RL- Right Lobe; mo.- months; AA- African American; ICC- Intrahepatic Cholangiocarcinoma; LELC- Lymphoepithelioma-like carcinoma components; OLT - orthotopic liver transplant; CT – Chemotherapy; op- operative; NA – Not Available.

*- Multiple Lesions

Supplementary Table 2: Summary of Immunohistochemical markers of LELCC ^[1-3, 5-7, 10-16, 18, 20, 21, 23, 24, 26-31, 33-35]^

| **No.** | **Study** | **Sex/Age** | **AE1/ A3** | **CK 7** | **CK 8** | **CK 18** | **CK 19** | **CK 20** | **Hep Par-1** | **p53** | **IDH1** | **LMP1** | **CEA** | **AFP** | **CDX2** | **CA-199** | **HER-2** | **TTF-1** | **MNF116** |
| --- | --- | --- | --- | --- | --- | --- | --- | --- | --- | --- | --- | --- | --- | --- | --- | --- | --- | --- | --- |
| 1 | **Current** | F/53 | NA | - | NA | - | + | + | NA | - | NA | NA | NA | NA | NA | - | + | NA | NA |
| 2 |  | M/41 | NA | + | NA | NA | + | - | NA | NA | NA | NA | NA | NA | - | NA | NA | - | NA |
| 3 | **Henderson-Jackson et al. ^[10]^** | F/63 | NA | + | NA | NA | + | + | - | NA | NA | NA | NA | NA | NA | NA | NA | NA | NA |
| 4 | **Zhang K et al. ^[1]^** | M/62 | NA | + | NA | + | + | NA | NA | NA | NA | NA | NA | - | NA | - | NA | NA | NA |
| 5 | **Binny et al. ^[7]^** | F/30 | NA | NA | NA | NA | + | NA | - | - | - | NA | NA | NA | NA | NA | NA | NA | + |
| 6 |  | M/71 | NA | NA | NA | NA |  | NA | NA | - | - | NA | NA | NA | NA | NA | NA | NA | NA |
| 7 |  | F/72 | NA | + | NA | NA | + | NA | - | NA | NA | NA | NA | NA | NA | NA | NA | NA | NA |
| 8 | **Wang et al. ^[3]^** | M/60 | NA | + | NA | NA | + | NA | - | NA | NA | NA | NA | NA | NA | NA | NA | NA | NA |
| 9 |  | M/53 | NA | + | NA | NA | + | NA | - | NA | NA | NA | NA | NA | NA | NA | NA | NA | NA |
| 10 |  | M/39 | NA | + | NA | NA | + | NA | - | NA | NA | NA | NA | NA | NA | NA | NA | NA | NA |
| 11 |  | F/43 | NA | + | NA | NA | + | NA | - | NA | NA | NA | NA | NA | NA | NA | NA | NA | NA |
| 12 |  | M/62 | NA | + | NA | NA | + | NA | - | NA | NA | NA | NA | NA | NA | NA | NA | NA | NA |
| 13 |  | F/65 | NA | + | NA | NA | + | NA | - | NA | NA | NA | NA | NA | NA | NA | NA | NA | NA |
| 14 |  | F/47 | NA | + | NA | NA | + | NA | - | NA | NA | NA | NA | NA | NA | NA | NA | NA | NA |
| 15 |  | M/52 | NA | + | NA | NA | + | NA | - | NA | NA | NA | NA | NA | NA | NA | NA | NA | NA |
| 16 |  | M/60 | NA | + | NA | NA | + | NA | - | NA | NA | NA | NA | NA | NA | NA | NA | NA | NA |
| 17 |  | M/61 | NA | + | NA | NA | + | NA | - | NA | NA | NA | NA | NA | NA | NA | NA | NA | NA |
| 18 |  | M/35 | NA | + | NA | NA | + | NA | - | NA | NA | NA | NA | NA | NA | NA | NA | NA | NA |
| 19 |  | M/48 | NA | + | NA | NA | + | NA | - | NA | NA | NA | NA | NA | NA | NA | NA | NA | NA |
| 20 |  | F/71 | NA | + | NA | NA | + | NA | - | NA | NA | NA | NA | NA | NA | NA | NA | NA | NA |
| 21 | **Gearty et al. ^[11]^** | F/28 | NA | - | NA | + | NA | NA | + | NA | NA | NA | NA | NA | NA | NA | NA | + | NA |
| 22 | **Adachi et al. ^[14]^** | M/64 | + | + | - | NA | NA | - | NA | NA | NA | - | - | - | NA | NA | NA | NA | NA |
| 23 | **Chen et al. ^[35]^** | F/67 | + | + | NA | NA | + | - | NA | NA | NA | NA | - | NA | NA | NA | NA | NA | NA |
| 24 |  | M/41 | + | + | NA | NA | + | - | NA | NA | NA | NA | - | NA | NA | NA | NA | NA | NA |
| 25 | **Ishida et al. ^[31]^** | M/68 | NA | + | NA | NA | + | - | - | + | NA | NA | NA | NA | NA | + | NA | NA | NA |
| 26 | **Kim et al. ^[30]^** | M/64 | - | NA | NA | NA | + | NA | NA | NA | NA | NA | - | - | NA | NA | NA | NA | NA |
| 27 | **Labgaa et al. ^[6]^** | M/58 | + | + | NA | NA | + | NA | - | NA | NA | NA | NA | - | NA | NA | NA | NA | NA |
| 28 | **Lee ^[29]^** | M/79 | + | + | NA | NA | + | - | - | + | NA | NA | - | - | NA | NA | NA | NA | NA |
| 29 | **Liao et al. ^[28]^** | F/35 | + | NA | NA | NA | NA | NA | NA | NA | NA | NA | - | NA | NA | - | NA | NA | NA |
| 30 | **Ortiz et al. ^[23]^** | F/19 | + | NA | NA | NA | NA | NA | NA | + | NA | - | - | - | NA | NA | NA | NA | NA |
| 31 | **Vortmeyer et al. ^[18]^** | F/71 | + | NA | NA | NA | NA | NA | NA | NA | NA | + | - | NA | NA | NA | NA | NA | NA |
| 32 | **Chan et al. ^[12]^** | F/53 | NA | + | NA | NA | + | NA | - | - | NA | - | NA | NA | NA | NA | NA | NA | NA |
| 33 |  | F/40 | NA | + | NA | NA | + | NA | - | + | NA | - | NA | NA | NA | NA | NA | NA | NA |
| 34 |  | F/57 | NA | + | NA | NA | + | NA | - | + | NA | - | NA | NA | NA | NA | NA | NA | NA |
| 35 |  | F/56 | NA | + | NA | NA | + | NA | - | - | NA | - | NA | NA | NA | NA | NA | NA | NA |
| 36 |  | F/59 | NA | + | NA | NA | + | NA | - | - | NA | - | NA | NA | NA | NA | NA | NA | NA |
| 37 |  | F/45 | NA | + | NA | NA | + | NA | - | - | NA | - | NA | NA | NA | NA | NA | NA | NA |
| 38 |  | F/57 | NA | + | NA | NA | + | NA | - | + | NA | - | NA | NA | NA | NA | NA | NA | NA |
| 39 | **Aosasa** **et al. ^[13]^** | F/65 | NA | NA | NA | NA | + | NA | NA | NA | NA | NA | NA | - | NA | - | NA | NA | NA |
| 40 | **Ding et al. ^[34]^** | F/75 | NA | NA | NA | NA | - | NA | NA | NA | NA | NA | - | - | NA | - | NA | NA | NA |
| 41 | **Lin et al. ^[27]^** | F/61 | + | NA | NA | NA | NA | - | - | NA | NA | NA | + | NA | NA | + | NA | NA | NA |
| 42 |  | M/23 | + | NA | NA | NA | NA | - | - | NA | NA | NA | - | NA | NA | - | NA | NA | NA |
| 43 | **Huang et al. ^[33]^** | F/46 | NA | + | NA | NA | + | NA | - | NA | NA | NA | NA | NA | NA | NA | NA | NA | NA |
| 44 |  | F/60 | NA | + | NA | NA | + | NA | - | NA | NA | NA | NA | NA | NA | NA | NA | NA | NA |
| 45 |  | F/43 | NA | + | NA | NA | + | NA | - | NA | NA | NA | NA | NA | NA | NA | NA | NA | NA |
| 46 |  | F/43 | NA | + | NA | NA | + | NA | - | NA | NA | NA | NA | NA | NA | NA | NA | NA | NA |
| 47 |  | F/47 | NA | + | NA | NA | + | NA | - | NA | NA | NA | NA | NA | NA | NA | NA | NA | NA |
| 48 |  | F/62 | NA | + | NA | NA | + | NA | - | NA | NA | NA | NA | NA | NA | NA | NA | NA | NA |
| 49 |  | M/54 | NA | + | NA | NA | + | NA | - | NA | NA | NA | NA | NA | NA | NA | NA | NA | NA |
| 50 |  | F/54 | NA | + | NA | NA | + | NA | - | NA | NA | NA | NA | NA | NA | NA | NA | NA | NA |
| 51 |  | F/56 | NA | + | NA | NA | + | NA | - | NA | NA | NA | NA | NA | NA | NA | NA | NA | NA |
| 52 | **Yang et al. ^[2]^** | F/47 | NA | NA | NA | NA | NA | NA | NA | NA | NA | NA | - | - | NA | NA | NA | NA | NA |
| 53 |  | F/61 | NA | NA | NA | NA | NA | NA | NA | NA | NA | NA | - | - | NA | NA | NA | NA | NA |
| 54 |  | F/60 | NA | NA | NA | NA | NA | NA | NA | NA | NA | NA | - | - | NA | + | NA | NA | NA |
| 55 |  | F/30 | NA | NA | NA | NA | NA | NA | NA | NA | NA | NA | - | - | NA | NA | NA | NA | NA |
| 56 |  | F/53 | NA | NA | NA | NA | NA | NA | NA | NA | NA | NA | - | - | NA | NA | NA | NA | NA |
| 57 |  | F/33 | NA | NA | NA | NA | NA | NA | NA | NA | NA | NA | - | - | NA | NA | NA | NA | NA |
| 58 |  | F/43 | NA | NA | NA | NA | NA | NA | NA | NA | NA | NA | - | - | NA | + | NA | NA | NA |
| 59 |  | M/54 | NA | NA | NA | NA | NA | NA | NA | NA | NA | NA | - | - | NA | NA | NA | NA | NA |
| 60 |  | F/55 | NA | NA | NA | NA | NA | NA | NA | NA | NA | NA | - | - | NA | NA | NA | NA | NA |
| 61 |  | F/63 | NA | NA | NA | NA | NA | NA | NA | NA | NA | NA | - | - | NA | NA | NA | NA | NA |
| 62 |  | F/55 | NA | NA | NA | NA | NA | NA | NA | NA | NA | NA | - | - | NA | + | NA | NA | NA |
| 63 | **Tai et al. ^[21]^** | M/56 | NA | NA | NA | NA | + | NA | NA | NA | NA | NA | - | - | NA | - | NA | NA | NA |
| 64 | **Zhang et al. ^[15]^** | F/41 | + | + | NA | NA | + | - | NA | NA | NA | NA | NA | NA | NA | NA | NA | NA | NA |
| 65 | **Xiao et al. ^[16]^** | F/62 | NA | NA | + | + | + | - | NA | + | NA | - | + | NA | NA | NA | NA | NA | NA |
| 66 | **Nogami et al. ^[24]^** | F/27 | NA | + | NA | NA | + | NA | NA | NA | NA | NA | NA | NA | NA | NA | NA | NA | NA |
| 67 | **Li et al. ^[5]^** | M/55 | NA | + | NA | NA | + | NA | - | NA | NA | NA | NA | NA | NA | NA | NA | NA | NA |
| 68 | **Ling et al. ^[26]^** | M/64 | NA | + | NA | NA | + | - | - | NA | NA | NA | - | - | NA | - | NA | NA | NA |
| 69 |  | M/40 | NA | + | NA | NA | + | - | - | NA | NA | NA | - | - | NA | + | NA | NA | NA |
| 70 | **Tan et al. ^[20]^** | M/22 | NA | NA | NA | NA | NA | NA | NA | NA | NA | NA | NA | NA | + | NA | NA | NA | NA |

M- Male; F- Female; CK- Cytokeratin; IDH1- Isocitrate dehydrogenase-1; LMP1- Latent membrane protein-1; CEA- Carcinoembryonic antigen; AFP- Alpha-fetoprotein; CDX-2- Homeobox protein; CA- Cancer antigen; HER-2- Human epidermal growth factor receptor 2; TTF-1- Thyroid transcription factor-1; NA- Not Available

Supplementary Table 3: Summary of Clinical imaging features of LELCC ^[1, 2, 5, 6, 8-11, 13, 15, 20, 21, 23, 24, 26, 28, 29, 32, 34, 35]^

| No. | Author | Sex/Age | Clinical Imaging features | | |
| --- | --- | --- | --- | --- | --- |
|  |  |  | Computed tomography (CT) | Magnetic resonance imaging (MRI) | Ultrasound (US) |
| 1 | **Current Series** | F/53 | Heterogenous enhancement, enhanced nodules |  | Hyperechoic |
| 2 |  | M/41 | Heterogenous enhancement, low attenuated shadow with irregular boundaries |  | Mixed echoic |
| 3 | **Yang et al. ^[2]^** | F/47 |  | Homogeneously marked enhancement in arterial phase, gradual washout, delayed central scar enhancement |  |
| 4 |  | F/61 |  | Homogeneously marked enhancement in arterial phase, gradual washout, delayed central scar enhancement |  |
| 5 |  | F/60 |  | Homogeneously marked enhancement in arterial phase, gradual washout, delayed central scar enhancement |  |
| 6 |  | F/30 |  | Cyst present, homogenous moderate enhancement in arterial phase, gradual washout, delayed central patchy enhancement, hypointense in hepatobiliary phase |  |
| 7 |  | F/53 | Moderate homogenous enhancement in arterial phase, gradual washout, delayed central patchy enhancement | Homogenous moderate enhancement in arterial phase, gradual washout, delayed central patchy enhancement, hypointense in hepatobiliary phase |  |
| 8 |  | F/33 | Mild homogenous advancements in arterial Phase, gradual washout |  |  |
| 9 |  | F/43 | Heterogenous, Irregular moderate peripheral enhancement with centripetal enhancement |  |  |
| 10 |  | M/54 | Homogenous mild advancements in arterial Phase, gradual washout, incomplete delayed pseudo capsule enhancement |  |  |
| 11 |  | F/55 | Heterogenous, Irregular peripheral enhancement with centripetal enhancement, incomplete delayed pseudo capsule enhancement |  |  |
| 12 |  | F/63 | Heterogenous, irregular peripheral enhancement with centripetal enhancement, incomplete delayed pseudo capsule enhancement |  |  |
| 13 |  | F/55 |  | Heterogenous, Irregular peripheral enhancement with centripetal enhancement, incomplete delayed pseudo capsule enhancement |  |
| 14 | **Hsu et al. ^[9]^** | F/47 | Hypovascular |  |  |
| 15 | **Ortiz et al. ^[23]^** | F/19 | Hypovascular |  |  |
| 16 | **Jeng et al. ^[8]^** | F/67 | Hypodense at UP |  |  |
| 17 |  | M/50 | Hypodense at UP |  |  |
| 18 |  | F/50 |  |  | Hyperechoic |
| 19 | **Chen et al. ^[35]^** | F/67 |  |  | Mixed Echoic |
| 20 |  | F/41 |  |  | Hyperechoic |
| 21 | **Henderson-Jackson et al. ^[10]^** | F/63 | Hypodense at UP |  |  |
| 22 | **Hur et al. ^[32]^** | F/57 |  | Centrifugal enhancement at Venous phase |  |
| 23 | **Lee ^[29]^** | M/79 | Hypervascular |  |  |
| 24 | **Liao et al. ^[28]^** | F/35 | Hypodense at UP, mild enhancement at early arterial phase | T1 homogenous hypointense, T2 hyperintense, early enhancement at arterial phase, washout at venous phase with central scar delayed enhancement | Hyperechoic |
| 25 | **Tai et al. ^[21]^** | M/56 | Delayed central contrast enhancement at venous phase |  |  |
| 26 | **Labgaa et al. ^[6]^** | M/58 | Enhanced at arterial phase, washed out at venous phase |  |  |
| 27 | **Gearty et al. ^[11]^** | F/28 | Heterogeneously enhanced with necrosis at venous phase |  |  |
| 28 | **Aosasa** **et al. ^[13]^** | F/64 | Hypodense at UP, slightly enhanced at arterial phase | Hypointense on T1W1, Hyperintense on T2W1 and DWI |  |
| 29 | **Ding et al. ^[34]^** | M/75 | Low attenuation at UP, Moderate enhancement at arterial phase | T1 hypointense, T2 Hyperintense, Marked enhancement at arterial phase. |  |
| 30 | **Li et al. ^[5]^** | M/55 |  | Hypointense on T1-weighted images, hyperintense on T2-weighted images, heterogenic perfusion defect in the hepatobiliary phase | Hyperenhancement in the arterial phase with rapid washout, venous and delay phases showed hypoenhancement (CEUS) |
| 31 | **Ling et al. ^[26]^** | M/64 | Hypodense nodule with unclear margin, had isoenhancement in the arterial phase and slight hypoenhancement in the venous phase |  | Slightly heterogeneous hypoechoic nodule |
| 32 |  | M/40 | Hypodense mass with peripheral rim-shape hyperdense and irregular margin. In the arterial phase, the centripetal part was isoenhanced. The periphery of the mass showed slight hyperenhancement, but the center washed out to hypoenhancement in the portal phase. |  | Heterogeneous hypoechoic, homogeneous hyperenhancement in the arterial phase (Grey US). In the venous phase and late phase, the center enhancement of the mass washed out gradually, presenting hypoenhancement, while the periphery demonstrated rim-like slight hyperenhancement. (CEUS) |
| 33 | **Tan et al. ^[20]^** | M/22 |  | Central T2 hyperintensity with peripheral rim enhancement |  |
| 34 | **Zhang et al. ^[15]^** | F/41 |  | Hypointensity on T1-weighted imaging and an irregular margin with hyperintensity on T2- weighted imaging | Heterogeneous arterial enhancement in the arterial phase, washout in the venous phase, and a hypoechoic nodule in the delayed phase |
| 35 | **Nogami et al. ^[24]^** | F/27 |  | Perfusion defect was shown in hepatobiliary phase | Hypoechoic, arterial phase showed hyper enhancement, and a perfusion defect in her Kupffer phase (CEUS) |
| 36 | **Zhang K et al. ^[1]^** | M/62 |  | Hypointense T1WI, enhancement in arterial phase |  |

M- Male; F- Female; CEUS - Contrast enhanced ultrasonography; UP- Unenhanced Phase

**References:**

1. Zhang, K., et al., *Lymphoepithelioma-like carcinoma in liver not associated with Epstein-Barr virus: a report of 3 cases and literature review.* 2020. **15**(1): p. 1-15.

2. Yang, Q., et al., *The CT and MRI features of primary intrahepatic lymphoepithelioma-like cholangiocarcinoma.* 2021. **216**(2): p. 393-402.

3. Wang, L., et al., *Programmed death-ligand 1 is upregulated in intrahepatic lymphoepithelioma-like cholangiocarcinoma.* 2016. **7**(43): p. 69749.

4. Sun, K., et al., *Clinicopathological features of 11 Epstein–Barr virus-associated intrahepatic cholangiocarcinoma at a single center in China.* 2016. **95**(40).

5. Li, X., et al., *Lymphoepithelioma‑Like Cholangiocarcinoma with Hepatitis C Virus Infection Treated by Microwave Ablation: A Literature Review and Case Report.* 2022. **14**: p. 2155.

6. Labgaa, I., A. Stueck, and S.C.J.T.A.j.o.p. Ward, *Lymphoepithelioma-like carcinoma in liver.* 2017. **187**(7): p. 1438-1444.

7. Khandakar, B., et al., *Lymphoepithelioma‐like neoplasm of the biliary tract with ‘probable low malignant potential’.* 2022. **80**(4): p. 720-728.

8. Jeng, Y.-M., C.-L. Chen, and H.-C.J.T.A.j.o.s.p. Hsu, *Lymphoepithelioma-like cholangiocarcinoma: an Epstein-Barr virus-associated tumor.* 2001. **25**(4): p. 516-520.

9. Hsu, H.-C., et al., *Clonal Epstein-Barr virus associated cholangiocarcinoma with lymphoepithelioma-like component.* 1996. **27**(8): p. 848-850.

10. Henderson-Jackson, E., et al., *Primary mixed lymphoepithelioma-like carcinoma and intra-hepatic cholangiocarcinoma: a case report and review of literature.* 2010. **3**(7): p. 736.

11. Gearty, S.V., et al., *An EBV+ lymphoepithelioma-like cholangiocarcinoma in a young woman with chronic hepatitis B.* 2019. **12**(7): p. e229520.

12. Chan, A.W., et al., *E pstein–B arr virus‐associated lymphoepithelioma‐like cholangiocarcinoma: a rare variant of intrahepatic cholangiocarcinoma with favourable outcome.* 2014. **65**(5): p. 674-683.

13. Aosasa, S., et al., *Intrahepatic cholangiocarcinoma with lymphoepithelioma-like carcinoma components not associated with Epstein-Barr virus: report of a case.* 2015. **100**(4): p. 689-695.

14. Adachi, S., O. Morimoto, and T.J.P.i. Kobayashi, *Lymphoepithelioma‐like cholangiocarcinoma not associated with EBV.* 2008. **58**(1): p. 69-74.

15. Zhang, J.-W., et al., *Surgical treatment for metastasis from lymphoepithelioma-like cholangiocarcinoma in the liver: A case report.* 2018. **97**(19).

16. Xiao, P., et al., *Epstein–Barr virus-associated intrahepatic cholangiocarcinoma bearing an intense lymphoplasmacytic infiltration.* 2012. **65**(6): p. 570-573.

17. Wei, J., et al., *Lymphoepithelioma-like hepatocellular carcinoma without Epstein-Barr virus infection: a case report and a review of the literature.* 2015. **58**(4): p. 550.

18. Vortmeyer, A.O., et al., *Hepatobiliary lymphoepithelioma-like carcinoma associated with Epstein-Barr virus.* 1998. **109**(1): p. 90-95.

19. Tsai, J.-H., et al., *Lymphoepithelioma-like intrahepatic cholangiocarcinoma is a distinct entity with frequent pTERT/TP53 mutations and comprises 2 subgroups based on epstein-barr virus infection.* 2021. **45**(10): p. 1409-1418.

20. Tan, N.Y., et al., *Metastatic Epstein-Barr virus-positive Lymphoepithelioma-like Cholangiocarcinoma in a young man with ulcerative colitis.* 2017. **112**(3): p. 518-520.

21. Tai, Y., R. Ngan, and S.J.H.K.J.R. Ting, *Coexistence of two Epstein-Barr virus–associated malignancies: lymphoepithelioma-like cholangiocarcinoma in a patient with a history of undifferentiated nasopharyngeal carcinoma.* 2015. **18**: p. 311-315.

22. Szekely, E.J.T.A.j.o.s.p., *Lymphoepithelioma-like cholangiocarcinoma (LELC) not associated with Epstein-Barr virus.* 2001. **25**(11): p. 1464-1465.

23. Ortiz, M.R., et al., *Epstein-Barr virus-associated cholangiocarcinoma with lymphoepithelioma-like component.* 2000. **8**(4): p. 347-351.

24. Nogami, A., et al., *Lymphoepithelioma-like cholangiocarcinoma with Epstein–Barr virus infection treated by radiofrequency ablation.* 2021. **14**(2): p. 638-644.

25. Min, H.S., E. Shin, and J.J.J.T.K.J.o.H. Jang, *Carcinoma with predominant lymphoid stroma in hepatobiliary system--report of 2 cases.* 2007. **13**(2): p. 222-227.

26. Ling, W., et al., *Ultrasonographic findings of intrahepatic lymphoepithelioma-like cholangiocarcinoma associated with Epstein–Barr virus: Two cases report.* 2019. **98**(3).

27. Lin, A., et al., *Lymphoepithelioma‐Like Carcinomas: A Rare Variant of Cholangiocarcinoma.* 2020. **72**(1): p. 353-355.

28. Liao, T.-C., et al., *Lymphoepithelioma-like cholangiocarcinoma: a mimic of hepatocellular carcinoma on imaging features.* 2015. **21**(13): p. 4089.

29. Lee, W.J.C.r.i.o., *Intrahepatic lymphoepithelioma-like cholangiocarcinoma not associated with epstein-barr virus: a case report.* 2011. **4**(1): p. 68-73.

30. Kim, Y.-B., et al., *Biliary lymphoepithelioma-like carcinoma not associated with Epstein-Barr virus.* 1999. **123**(5): p. 441-443.

31. Ishida, M., et al., *Non-Epstein-Barr virus associated lymphoepithelioma-like carcinoma of the inferior common bile duct.* 2011. **3**(7): p. 111.

32. Hur, Y.H., et al., *Lymphoepithelioma‐like cholangiocarcinoma not associated with Epstein‐Barr virus.* 2011. **9**(81): p. 652-653.

33. Huang, Y.-H., et al., *Clinicopathologic features, tumor immune microenvironment and genomic landscape of Epstein-Barr virus-associated intrahepatic cholangiocarcinoma.* 2021. **74**(4): p. 838-849.

34. Ding, Y., et al., *Lymphoepithelioma-like intrahepatic cholangiocarcinoma with Epstein-Barr virus infection: report of a rare case.* 2019. **7**(18).

35. Chen, T.-C., K.-F. Ng, and T.-t.J.M.P. Kuo, *Intrahepatic cholangiocarcinoma with lymphoepithelioma-like component.* 2001. **14**(5): p. 527-532.
